# The Reciprocal Relationship between Osteoporosis and Renal Stones

**DOI:** 10.3390/jcm11226614

**Published:** 2022-11-08

**Authors:** So Young Kim, Juyong Chung, Doo Sik Park, Dae Myoung Yoo, Woo Jin Bang, Hyo Geun Choi

**Affiliations:** 1 Department of Otorhinolaryngology—Head & Neck Surgery, CHA Bundang Medical Center, CHA University, Seongnam 13496, Korea; 2 Department of Otorhinolaryngology—Head and Neck Surgery, Wonkwang University School of Medicine, Iksan 54538, Korea; 3 Department of Otorhinolaryngology—Head & Neck Surgery, Hallym University College of Medicine, Anyang 14068, Korea; 4 Hallym Data Science Laboratory, Hallym University College of Medicine, Anyang 14068, Korea; 5 Department of Urology, Hallym Sacred Heart Hospital, Hallym University College of Medicine, Anyang 14068, Korea

**Keywords:** urolithiasis, osteoporosis, risk factors, case–control studies, epidemiology

## Abstract

Previous studies have proposed an association between osteoporosis and renal stones. The current analyses intended to investigate the bidirectional relationship between osteoporosis and renal stones. The ≥40-year-old population in the National Health Insurance Service-Health Screening cohort (2002–2015) was analyzed. In study I, 67,811 patients with osteoporosis and 67,811 control I participants were matched. The hazard ratio (HR) of osteoporosis for renal stones was calculated using stratified Cox proportional hazard models. In study II, 25,261 patients with renal stones and 101,044 control II participants were matched. The HR of renal stones for osteoporosis was estimated using stratified Cox proportional hazard models. In study I, 3.4% (2276/67,811) of osteoporosis patients and 2.5% (1696/67,811) of control I participants had renal stones. Osteoporosis patients had a 1.36 times higher HR for renal stones than control I participants (95% confidence intervals [CI] = 1.28–1.45). In study II, 9.2% (2319/25,261) of renal stone patients and 7.6% (7658/101,044) of control II participants had osteoporosis. Renal stone patients had a 1.26 times higher HR for osteoporosis than control II participants (95% CI = 1.21–1.32). Adults with osteoporosis had a higher risk of renal stones. Moreover, adults with renal stones had a higher risk of osteoporosis.

## 1. Introduction

Renal stones are a common disease affecting approximately 9.09% of the population in the US [1]. The prevalence of renal stones has been rising, probably attributed to lifestyle factors, such as obesity and nutritional intake [1]. Renal stones develop from mineral deposits that crystallize with organic components, mainly calcium oxalate stones [2]. In addition to lifestyle factors, chronic diseases, including diabetes, hypertension, and metabolic disorders, are risk factors for renal stones [2]. Moreover, osteoporosis has been proposed as a risk factor for renal stones [3]. Both renal stones and osteoporosis have a high incidence with aging and additional risks related to chronic diseases [3]. Thus, a review study posited osteoporosis and renal stones as one disease entity manifested as distinct clinical symptoms [3].

Osteoporosis is a prevalent metabolic disorder. The worldwide prevalence of osteoporosis was calculated to be approximately 18.3% in the general population and as high as 30% in postmenopausal women [4,5]. The reduced bone mineral density in adults can be caused by nutritional factors and chronic diseases disturbing mineral metabolisms, such as metabolic syndrome and chronic kidney disease [6]. Furthermore, a retrospective study demonstrated an increased risk of osteoporosis in patients with renal stones [7]. Because the potential risk of renal stones in patients with osteoporosis, as well as the possible risk of osteoporosis in patients with renal stones, have been suggested, a bidirectional association between renal stones and osteoporosis can be predicted. However, the reciprocal relationship between the two diseases has not been explored in previous studies.

We supposed that there may be a bidirectional association between renal stones and osteoporosis in an adult population. Because there may be genetic or congenital factors related to osteoporosis or renal stones in children or young populations, we excluded these populations from the current analyses. To test two directional relationships between renal stones and osteoporosis, two case–control studies were conducted with independently selected control populations. Moreover, because both age and sex are risk factors for both renal stones and osteoporosis, secondary analyses were performed according to age and sex subgroups.

## 2. Methods

### 2.1. Ethical Approval and Source of Data

The current study was approved by the ethics committee of Hallym University (23 October 2019). Written informed consent was waived by the ethics committee of Hallym University. The study participants were recruited from the National Health Insurance Service-Health Screening cohort (2002–2015) [8].

### 2.2. Classification of Diseases

The presence of osteoporosis and renal stones was classified based on the national health claim data. Among the patients with osteoporosis (ICD-10 codes: M80–M82), patients who visited the clinic 2 or more times with the health claim code of osteoporosis and who underwent bone density testing using X-ray or CT were enrolled. Among the patients with renal stones (N20), the patients who visited the clinic 2 or more times due to renal stones were included.

### 2.3. Study I

**Figure 1:**
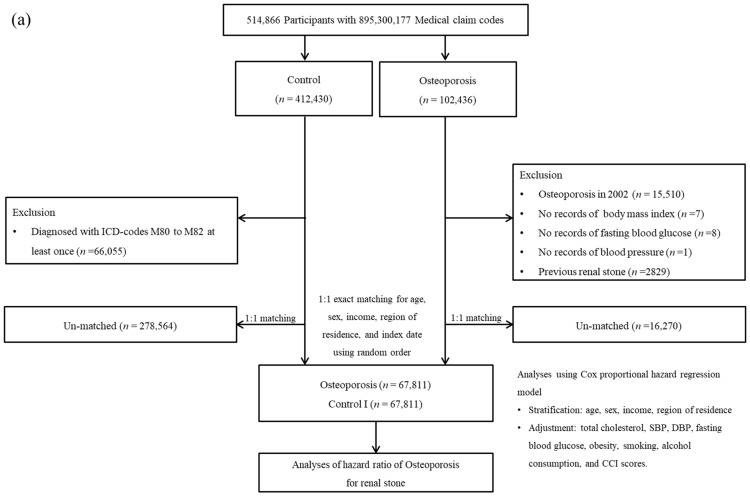

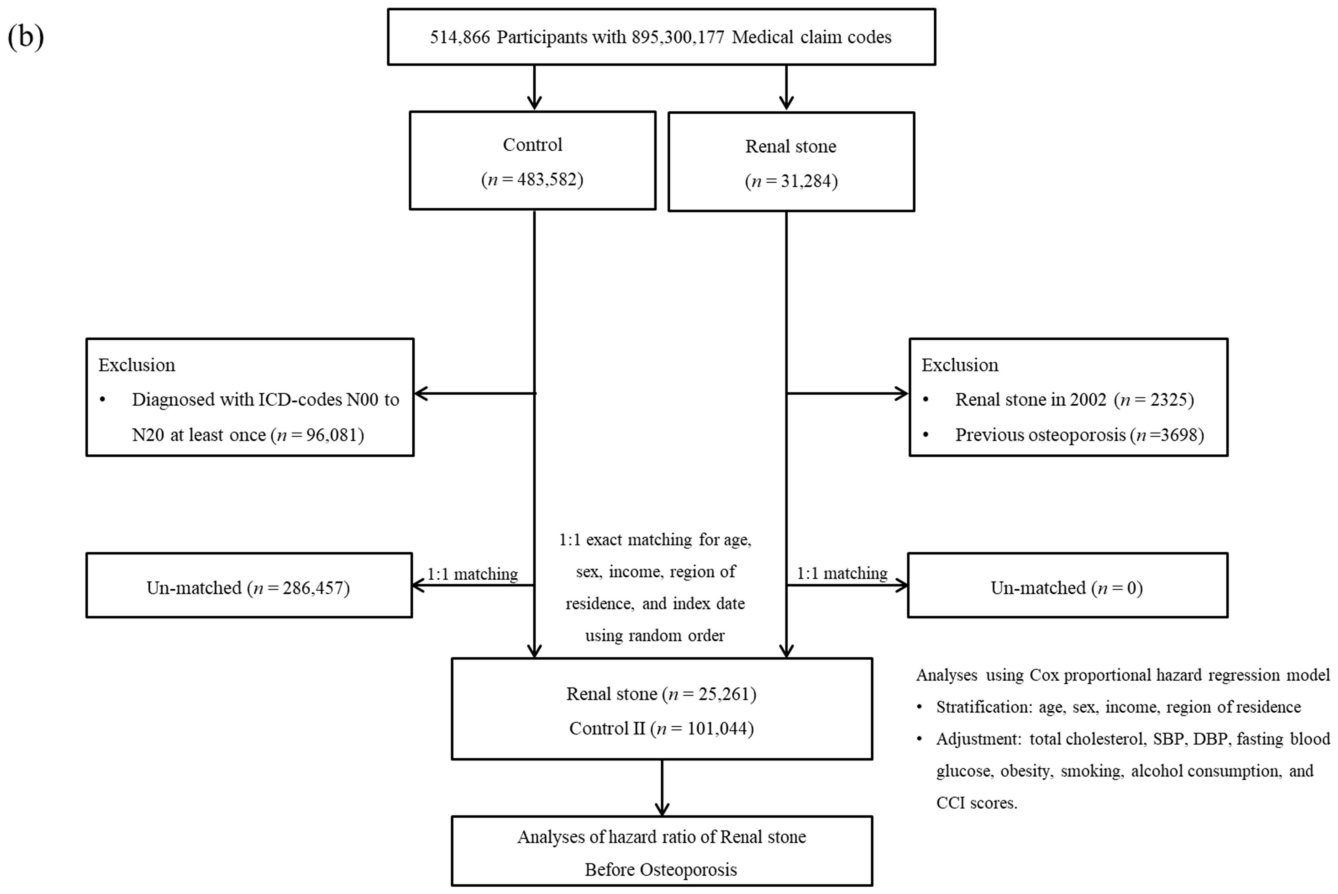
(**a**) A schematic illustration of the participant selection process that was used in the present study. Of a total of 514,866 participants, 67,811 osteoporosis participants were matched with 67,811 control participants for age, sex, income, and region of residence. (**b**) A schematic illustration of the participant selection process that was used in the present study. Of a total of 514,866 participants, 25,261 renal stone participants were matched with 101,044 control participants for age, sex, income, and region of residence.

Patients with osteoporosis from 2002 to 2019 were selected (*n* = 102,436). Among these osteoporosis patients, the patients who were diagnosed in 2002 were removed (*n* = 15,510). Control participants were selected among those who were not diagnosed with osteoporosis. Control participants were equalized with osteoporosis patients for age, sex, income, and region of residence. Ultimately, 67,811 osteoporosis participants and 67,811 control I participants were enrolled (Figure 1a).

### 2.4. Study II

Patients with renal stones from 2002 to 2019 were selected (*n* = 31,284). The renal stone patients who were initially diagnosed in 2002 were removed (*n* = 2325). Control participants were included who were not diagnosed with renal stones. The control participants were 1:4 equalized with renal stone patients for age, sex, income, and region of residence. Ultimately, 25,261 renal stone participants and 101,044 control II participants were included (Figure 1b).

### 2.5. Covariates

The information on age was provided as groups with 5-year intervals. Based on the national health claim classification of household income, the income group was divided into five groups. According to the house address registered in the national health claim data, urban and rural groups were classified. To measure comorbid conditions, the Charlson Comorbidity Index (CCI) was calculated based on the health claim codes. The history of smoking and alcohol consumption was surveyed during a national health check-up with a self-report questionnaire. Using BMI (body mass index, kg/m^2^), the BMI groups were classified [9]. The systolic blood pressure (SBP, mmHg), diastolic blood pressure (DBP, mmHg), fasting blood glucose (mg/dL), and total cholesterol (mg/dL) were collected during the national health check-up.

### 2.6. Statistical Analyses

The standardized difference in variables was calculated and compared between the study and control groups.

The hazard ratios (HRs) and 95% confidence intervals (CIs) were calculated using stratified Cox proportional hazard models. In study I, the HRs of osteoporosis for renal stones were estimated. In study II, the HRs of renal stones for osteoporosis were estimated. The variables of obesity, smoking, alcohol consumption, systolic blood pressure, diastolic blood pressure, fasting blood glucose, total cholesterol, CCI scores, and renal stones were adjusted. The cumulative incidence rates were calculated using the Kaplan–Meier curve and log-rank test.

The secondary analyses were conducted according to age, sex, income, and region. A *p* value < 0.05 was considered statistically significant. SAS version 9.4 (SAS Institute Inc., Cary, NC, USA) was used.

## 3. Results

A total of 3.4% (2276/67,811) of osteoporosis patients and 2.5% (1696/67,811) of control I participants had a history of renal stones (Table 1). The osteoporosis group demonstrated lower rates of obesity, current smoking, alcohol consumption, high blood pressure, high fasting blood glucose, and high total cholesterol levels than the control I group. The rate of high CCI scores was higher in the osteoporosis group than in the control I group.

**Figure 2:**
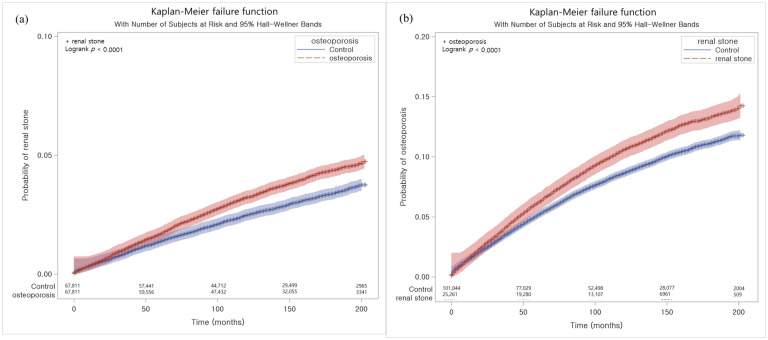
(**a**) The Kaplan–Meier method was applied in study I. The rate of renal stones was significantly higher in the osteoporosis group than in the control I group. (**b**) The Kaplan–Meier method was applied in study I. The rate of osteoporosis was significantly higher in the renal stone group than in the control II group.

The HR for renal stones was 1.36 times higher in the osteoporosis group than in the control I group in the adjusted model (95% CI = 1.28–1.45, *p* < 0.001, Table 2 and Figure 2a). The high HR for renal stones in patients with osteoporosis was consistent in all age, sex, income, and region of residence groups (all *p* < 0.001).

A total of 9.2% (2319/25,261) of the renal stone group and 7.6% (7658/101,044) of the control II group had a history of osteoporosis (Table 3). The rates of obesity, current smoking, alcohol consumption, high blood pressure, high fasting blood glucose, high total cholesterol, and high CCI score were higher in the renal stone group than in the control II group.

The HR for osteoporosis was 1.26 times higher in the osteoporosis group than in the control II group (95% CI = 1.21–1.32, *p* < 0.001, Table 4 and Figure 2b). The high HR for osteoporosis in patients with renal stones was consistent in all age, sex, income, and region of residence groups (all *p* < 0.001).

## 4. Discussion

Adults with osteoporosis showed a higher risk of renal stones. In addition, adults with renal stones had a higher risk of osteoporosis in this study. The reciprocal association between osteoporosis and renal stones was maintained in all subgroups according to age, sex, income, and region of residence. The current results add to previous knowledge on the association of osteoporosis with renal stones by analyzing bidirectional relationships using a large cohort population.

The patients with osteoporosis had a high risk of renal stones in our study I cohort. The risk of renal stones in patients with osteoporosis has been suggested [10,11,12,13]. It was demonstrated that the population with metabolic bone disease, largely osteoporosis, had a greater risk for renal stones [11]. The common risk factors for osteoporosis and renal stones can be linked to the subsequent occurrence of renal stones in osteoporosis patients. For instance, physical inactivity was noted as a common risk factor for osteoporosis and renal stones [11]. The underlying morbidity can increase the vulnerability to both osteoporosis and renal stones. In patients with ankylosing spondylitis, low-bone mineral density was related to the presence of renal stones [12]. Low-bone mineral density and activated resorptive bone metabolism can systematically influence the risk of calcification in multiple organs. Low-bone mineral density and increased bone resorptive markers were related to mitral annular calcification and renal stones [13].

In addition, it was supposed that calcium supplements in patients with osteoporosis can increase the risk of subsequent renal stones. However, many previous studies described no additional risk of renal stones associated with high calcium intake [14]. On the other hand, high calcium intake was related to a reduced risk of renal stones [14]. A review study that analyzed eight randomized clinical trials and two cohort studies reported no relation between calcium medication in osteoporosis patients and the risk of renal stone occurrence [10]. The reason for this inverse association was explained by the reduced urinary oxalate concentration. Because oxalate is a known risk factor for renal stone formation, a reduced oxalate concentration may impede renal stone formation. In line with this, some previous reports questioned the risk of renal stones in patients with osteoporosis [10,11]. Although the common risk factors and underlying pathophysiology of osteoporosis and renal stones have been suggested, the risk of osteoporosis was not always linked with the risk of renal stones. For instance, the occurrence of osteoporosis was high in patients with hyperparathyroidism, but the rate of renal stones was not high in these patients [11]. It can be hypothesized that the hormonal disturbance related to osteoporosis may not be relevant to the occurrence of renal stones. The irrelevance of the risk of renal stones with pre-existing osteoporosis may be attributed to the fact that the development of detectable renal stones can take a long time, with a few years; thus, the increased risk of renal stones cannot be detected without a sufficiently long-term follow-up duration.

The patients with renal stones had an elevated risk of osteoporosis in our study II cohorts. A few prior analyses suggested a greater risk of osteoporosis in patients with renal stones [7,15,16,17,18,19,20]. A meta-analysis described a greater rate of osteoporosis in patients with renal stones (odds ratio = 4.12, 95% CI = 3.99–4.26) [15]. In a case–control study using health claim data, patients with renal stones had a higher incidence of osteoporosis than control participants (adjusted HR = 1.34, 95% CI = 1.19–1.79) [7]. Subsequent osteoporosis has been detected in 20% of patients with renal stones by DXA screening [16]. The increased occurrence of osteoporosis related to renal stones was evident for the various locations of renal stones, including the kidney, ureter, bladder, and unspecified locations [18]. Renal stones can be an indicator of bone calcium efflux [21]. In patients with renal stones and low bone mineral density, the rate of fasting hypercalciuria was high [21]. It was estimated that as many as 35% of patients with osteopenia demonstrated fasting calciuria [21]. However, in patients with renal calcium stone diseases, renal calcium leakage is not observed commonly in 24 h urine collection. Thus, additional mechanisms can contribute to the link between renal stones and osteoporosis. For example, restricted calcium intake can be associated with the risk of osteoporosis in patients with renal stones. In addition, underlying morbidities in patients with renal stones can predispose them to the occurrence of osteoporosis.

In our cohort, the risk of osteoporosis was high in both the young and old populations. In addition, the reciprocal associations between osteoporosis and renal stones were valid in both sexes. However, in a previous study, older patients with renal stones (>60 years old) demonstrated a higher rate of osteoporosis than younger patients with renal stones [17]. Because the elderly population has prevalent morbidities, including osteoporosis and renal stones, the relationship between osteoporosis and renal stones can be stronger than that in younger populations. According to sex, because osteoporosis is prevalent in postmenopausal women and the pathophysiology of osteoporosis is different in these populations, the relationship of osteoporosis with renal stones can be distinguished according to sex. In the present study, the large study population may enhance the statistical power, in that a significant association can exist in all age and sex subgroups. Further study is warranted to determine the age- and sex-specific relationship between osteoporosis and renal stones.

The present study used a large population cohort. A large number of participants enhanced the statistical power of the present analyses. The reciprocal association between osteoporosis and renal stones may be clinically applied to the early prevention and diagnosis of renal stones in patients with osteoporosis and osteoporosis in patients with renal stones. The control participants can be randomly selected to minimize bias during this process. In addition, numerous covariables were considered to attenuate the potential confounder effects. However, this study was based on national health claim code data in that the type or severity of osteoporosis and renal stones could not be assessed. The composition of renal stones, such as calcium, cystine, struvite, and uric acid, cannot be differentiated in the present study. Although the impacts of dietary factors on renal stones have been controversial, dietary intake can influence the risk of renal stones or osteoporosis. For instance, patients with renal stones can limit their intake of calcium-rich food sources such as dairy products. These factors were not available in the current cohort data. Therefore, the cause–effect relationship cannot be determined in the current study. The diagnosis of osteoporosis and renal stones was determined using health claim code data in the current cohort. Thus, patients who did not visit clinics can be missed in this cohort. The medication or surgical intervention for renal stones was not accessed in this study. Future studies with specified types of osteoporosis and renal stones can reveal the detailed links between the two diseases. In addition, the detailed mechanism of the link between the two diseases should be elaborated in further studies.

## 5. Conclusions

The presence of osteoporosis increased the susceptibility to renal stones in the adult population. In addition, the presence of renal stones elevated the vulnerability to osteoporosis. The reciprocal relationship between osteoporosis and renal stones needs to be considered when managing patients with osteoporosis or renal stones.

## Figures and Tables

**Table 1:** General Characteristics of Participants.

Characteristics	Total Participants		
	Osteoporosis (*n*, %)	Control (*n*, %)	Standardized Difference
Total number	67,811 (100.0)	67,811 (100.0)	
Age (years old)			0.00
40–44	1025 (1.5)	1025 (1.5)	
45–49	5110 (7.5)	5110 (7.5)	
50–54	12,051 (17.8)	12,051 (17.8)	
55–59	15,211 (22.4)	15,211 (22.4)	
60–64	13,563 (20.0)	13,563 (20.0)	
65–69	7263 (10.7)	7263 (10.7)	
70–74	6944 (10.2)	6944 (10.2)	
75–79	4458 (6.6)	4458 (6.6)	
80–84	1826 (2.7)	1826 (2.7)	
85+	360 (0.5)	360 (0.5)	
Sex			0.00
Male	12,306 (18.2)	12,306 (18.2)	
Female	55,505 (81.9)	55,505 (81.9)	
Income			0.00
1 (lowest)	12,855 (19.0)	12,855 (19.0)	
2	10,246 (15.1)	10,246 (15.1)	
3	11,083 (16.3)	11,083 (16.3)	
4	13,786 (20.3)	13,786 (20.3)	
5 (highest)	19,841 (29.3)	19,841 (29.3)	
Region of residence			0.00
Urban	28,576 (42.1)	28,576 (42.1)	
Rural	39,235 (57.9)	39,235 (57.9)	
Obesity †			0.18
Underweight	2400 (3.5)	1632 (2.4)	
Normal	27,361 (40.4)	23,234 (34.3)	
Overweight	17,731 (26.2)	17,716 (26.1)	
Obese I	18,530 (27.3)	22,309 (32.9)	
Obese II	1789 (2.6)	2920 (4.3)	
Smoking status			0.5
Nonsmoker	60,277 (88.9)	59,566 (87.8)	
Past smoker	3451 (5.1)	3572 (5.3)	
Current smoker	4083 (6.0)	4673 (6.9)	
Alcohol consumption			0.05
<1 time a week	56,281 (83.0)	55,306 (81.6)	
≥1 time a week	11,530 (17.0)	12,505 (18.4)	
Systolic blood pressure			0.13
<120 mmHg	23,007 (33.9)	20,459 (30.2)	
120–139 mmHg	30,208 (44.6)	29,831 (44.0)	
≥140 mmHg	14,596 (21.5)	17,521 (25.8)	
Diastolic blood pressure			0.11
<80 mmHg	34,028 (50.2)	31,177 (46.0)	
80–89 mmHg	22,243 (32.8)	22,738 (33.5)	
≥90 mmHg	11,540 (17.0)	13,896 (20.5)	
Fasting blood glucose			0.13
<100 mg/dL	47,274 (69.7)	43,952 (64.8)	
100–125 mg/dL	16,240 (24.0)	17,471 (25.8)	
≥126 mg/dL	4297 (6.3)	6388 (9.4)	
Total cholesterol			0.04
<200 mg/dL	33,542 (49.5)	32,588 (48.1)	
200–239 mg/dL	23,440 (34.6)	23,523 (34.7)	
≥240 mg/dL	10,829 (16.0)	11,700 (17.3)	
CCI score			0.13
0	37,311 (55.0)	40,827 (60.2)	
1	12,535 (18.5)	10,550 (15.6)	
≥2	17,965 (26.5)	16,434 (24.2)	
Renal stone	2276 (3.4)	1696 (2.5)	0.05

Abbreviation: CCI, Charlson comorbidity index; † Obesity (BMI, body mass index, kg/m^2^) was categorized as <18.5 (underweight), ≥18.5 to <23 (normal), ≥23 to <25 (overweight), ≥25 to <30 (obese I), and ≥30 (obese II).

**Table 2:** Crude and adjusted hazard ratios of osteoporosis for renal stones by subgroup according age, sex, income, and region.

Independent Variables	IR per 1000 Person-Year	IRD per 1000 Person-Years (95% CI)	Hazard Ratios for Renal Stone (95% Confidence Interval)			
			Crude ^†^	*p* Value	Adjusted ^†,‡^	*p* Value
Total participants (*n* = 135,622)						
Osteoporosis	3.2	0.70 (0.52 to 0.88)	1.30 (1.22 to 1.39)	<0.001 *	1.36 (1.28 to 1.45)	<0.001 *
Control	2.5		1		1	
Age < 60 (*n* =66,794)						
Osteoporosis	3.7	0.93 (0.68 to 1.19)	1.34 (1.24 to 1.46)	<0.001 *	1.41 (1.29 to 1.53)	<0.001 *
Control	2.7		1		1	
Age ≥ 60 (*n* = 68,828)						
Osteoporosis	2.3	−0.46 (−0.70 to −0.21)	1.24 (1.12 to 1.37)	<0.001 *	1.30 (1.17 to 1.44)	<0.001 *
Control	2.7		1		1	
Men (*n* = 24,612)						
Osteoporosis	4.0	0.54 (−0.04 to 1.12)	1.17 (1.00 to 1.37)	0.044 *	1.37 (1.15 to 1.64)	0.001 *
Control	3.4		1		1	
Women (*n* = 111,010)						
Osteoporosis	3.1	0.72 (0.53 to 0.91)	1.33 (1.24 to 1.42)	<0.001 *	1.36 (1.27 to 1.46)	<0.001 *
Control	2.4		1		1	
Low income (*n* = 68,368)						
Osteoporosis	3.1	0.77 (0.52 to 1.01)	1.35 (1.24 to 1.48)	<0.001 *	1.38 (1.26 to 1.51)	<0.001 *
Control	2.4		1		1	
High income (*n* = 67,254)						
Osteoporosis	3.3	0.63 (0.37 to 0.89)	1.25 (1.15 to 1.37)	<0.001 *	1.29 (1.18 to 1.41)	<0.001 *
Control	2.7		1		1	
Urban residents (*n* = 57,152)						
Osteoporosis	3.2	0.67 (0.40 to 0.94)	1.33 (1.24 to 1.42)	<0.001 *	1.31 (1.19 to 1.44)	<0.001 *
Control	2.5		1		1	
Rural residents (*n* = 78,470)						
Osteoporosis	0.1	−0.18 (−0.23 to −0.12)	1.28 (1.16 to 1.41)	<0.001 *	1.35 (1.24 to 1.47)	<0.001 *
Control	0.2		1		1	

Abbreviations; IR, incidence rate; IRD, incidence rate difference; * Stratified Cox proportional hazard regression model, Significance at *p* < 0.05; † Models were stratified by age, sex, income, and region of residence. ‡ The model was adjusted for obesity, smoking, alcohol consumption, systolic blood pressure, diastolic blood pressure, fasting blood glucose, total cholesterol, and CCI scores.

**Table 3:** General Characteristics of Participants.

Characteristics	Total Participants		
	Renal Stone (*n*, %)	Control (*n*, %)	Standardized Difference
Total number	25,261 (100.0)	101,044 (100.0)	
Age (years old)			0.00
40–44	1162 (4.6)	4648 (4.6)	
45–49	3498 (13.9)	13,992 (13.9)	
50–54	5134 (20.3)	20,536 (20.3)	
55–59	5557 (22.0)	22,228 (22.0)	
60–64	4289 (17.0)	17,156 (17.0)	
65–69	2738 (10.8)	10,952 (10.8)	
70–74	1651 (6.5)	6604 (6.5)	
75–79	857 (3.4)	3428 (3.4)	
80–84	289 (1.1)	1156 (1.1)	
85+	86 (0.3)	344 (0.3)	
Sex			0.00
Male	18,200 (72.1)	72,800 (72.1)	
Female	7061 (28.0)	28,244 (28.0)	
Income			0.00
1 (lowest)	3428 (13.6)	13,712 (13.6)	
2	2950 (11.7)	11,800 (11.7)	
3	3916 (15.5)	15,664 (15.5)	
4	5528 (21.9)	22,112 (21.9)	
5 (highest)	9439 (37.4)	37,756 (37.4)	
Region of residence			0.00
Urban	11,219 (44.4)	44,876 (44.4)	
Rural	14,042 (55.6)	56,168 (55.6)	
Obesity †			0.18
Underweight	309 (1.2)	2063 (2.0)	
Normal	6969 (27.6)	34,846 (34.5)	
Overweight	7322 (29.0)	28,527 (28.2)	
Obese I	9729 (38.5)	32,905 (32.6)	
Obese II	932 (3.7)	2703 (2.7)	
Smoking status			0.14
Nonsmoker	15,012 (59.4)	67,025 (66.3)	
Past smoker	4800 (19.0)	16,222 (16.1)	
Current smoker	5449 (21.6)	17,797 (17.6)	
Alcohol consumption			0.07
<1 time a week	15,185 (60.1)	64,127 (63.5)	
≥1 time a week	10,076 (39.9)	36,917 (36.5)	
Systolic blood pressure			0.02
<120 mmHg	7180 (28.4)	30,437 (30.1)	
120–139 mmHg	13,006 (51.5)	50,408 (49.9)	
≥140 mmHg	5075 (20.1)	20,199 (20.0)	
Diastolic blood pressure			0.03
<80 mmHg	10,932 (43.3)	45,395 (44.9)	
80–89 mmHg	9652 (38.2)	37,229 (36.8)	
≥90 mmHg	4677 (18.5)	18,420 (18.2)	
Fasting blood glucose			0.04
<100 mg/dL	14,854 (58.8)	61,919 (61.3)	
100–125 mg/dL	7698 (30.5)	29,732 (29.4)	
≥126 mg/dL	2709 (10.7)	9393 (9.3)	
Total cholesterol			0.04
<200 mg/dL	13,284 (52.6)	55,208 (54.6)	
200–239 mg/dL	8454 (33.5)	33,059 (32.7)	
≥240 mg/dL	3523 (14.0)	12,777 (12.6)	
CCI score			0.15
0	15,210 (60.2)	67,732 (67.0)	
1	4452 (17.6)	14,943 (14.8)	
≥2	5599 (22.2)	18,369 (18.2)	
Osteoporosis	2319 (9.2)	7658 (7.6)	0.06

Abbreviation: CCI, Charlson comorbidity index; † Obesity (BMI, body mass index, kg/m^2^) was categorized as <18.5 (underweight), ≥18.5 to <23 (normal), ≥23 to <25 (overweight), ≥25 to <30 (obese I), and ≥30 (obese II).

**Table 4:** Crude and adjusted hazard ratios of renal stones for osteoporosis stratified by age, sex, income, and region.

Independent Variables	IR per 1000 Person-Year	IRD per 1000 Person-Years (95% CI)	Hazard Ratios for Osteoporosis (95% Confidence Interval)			
			Crude ^†^	*p* Value	Adjusted ^†,‡^	*p* Value
Total participants (*n* = 126,305)						
Renal stone	11.3	1.98 (1.50 to 2.46)	1.24 (1.18 to 1.29)	<0.001 *	1.26 (1.21 to 1.32)	<0.001 *
Control	9.3		1		1	
Age < 60 (*n* = 76,755)						
Renal stone	7.8	1.55 (1.09 to 2.01)	1.28 (1.20 to 1.37)	<0.001 *	1.31 (1.23 to 1.40)	<0.001 *
Control	6.2		1		1	
Age ≥ 60 (*n* = 49,550)						
Renal stone	20.4	3.00 (1.77 to 4.22)	1.19 (1.12 to 1.28)	<0.001 *	1.22 (1.14 to 1.30)	<0.001 *
Control	17.4		1		1	
Men (*n* = 91,000)						
Renal stone	3.5	0.80 (0.50 to 1.10)	1.29 (1.17 to 1.42)	<0.001 *	1.38 (1.25 to 1.53)	0.001 *
Control	2.7		1		1	
Women (*n* = 35,305)						
Renal stone	34.4	6.41 (4.77 to 8.05)	1.22 (1.16 to 1.29)	<0.001 *	1.24 (1.17 to 1.30)	<0.001 *
Control	28.0		1		1	
Low income (*n* = 51,470)						
Renal stone	14.3	2.92 (2.07 to 3.76)	1.29 (1.21 to 1.38)	<0.001 *	1.32 (1.23 to 1.41)	<0.001 *
Control	11.4		1		1	
High income (*n* = 74,835)						
Renal stone	9.3	1.39 (0.82 to 1.95)	1.19 (1.11 to 1.27)	<0.001 *	1.21 (1.14 to 1.30)	<0.001 *
Control	8.0		1		1	
Urban residents (*n* = 56,095)						
Renal stone	10.6	2.31 (1.64 to 2.98)	1.30 (1.21 to 1.40)	<0.001 *	1.33 (1.24 to 1.43)	<0.001 *
Control	8.3		1		1	
Rural residents (*n* = 70,210)						
Renal stone	11.9	1.70 (1.02 to 2.37)	1.28 (1.12 to 1.26)	<0.001 *	1.22 (1.14 to 1.29)	<0.001 *
Control	10.2		1		1	

Abbreviations; IR, incidence rate; IRD, incidence rate difference; * Stratified Cox proportional hazard regression model, Significance at *p* < 0.05. † Models were stratified by age, sex, income, and region of residence. ‡ The model was adjusted for obesity, smoking, alcohol consumption, systolic blood pressure, diastolic blood pressure, fasting blood glucose, total cholesterol, and CCI scores.

## Data Availability

Releasing of the data by the researcher is not legally permitted. All data are available from the database of the Korea Centers for Disease Control and Prevention. The Korea Centers for Disease Control and Prevention allows data access, at a particular cost, for any researcher who promises to follow the research ethics. The data of this article can be downloaded from the website after agreeing to follow the research ethics.
